# Local Instantaneous Iron Loss Measurement in Grain-Oriented Electrical Steel

**DOI:** 10.3390/s26175380

**Published:** 2026-08-26

**Authors:** Yusuke Kawamura, Helmut Pfützner, Georgi Shilyashki

**Affiliations:** 1 Institute of Biomedical Electronics, TU Wien, 1040 Vienna, Austria; 2 Magnet Test Labs, Vienna Magnetics GmbH, 1020 Vienna, Austria; 3 Steel Research Laboratories, Nippon Steel Corporation, Chiba 293-8511, Japan

**Keywords:** grain-oriented electrical steel, local iron loss, instantaneous iron loss

## Abstract

Conventional iron loss measurement methods for grain-oriented electrical steel (GOES), typified by the single sheet tester (SST), evaluate the average iron loss over a large area during one excitation cycle. However, the spatial and temporal resolutions of these conventional methods are insufficient for evaluating local instantaneous iron loss associated with local magnetic domain structure changes occurring near grain boundaries in GOES. Therefore, this study proposes a novel measurement method that dramatically improves the spatial and temporal resolutions, enabling the measurement of local instantaneous iron loss. To validate the proposed method, the local instantaneous iron loss at the same location was compared before and after magnetic domain refinement.

## 1. Introduction

Grain-oriented electrical steel (GOES) is a soft magnetic material widely used for transformer cores [1,2]. Iron loss is one of the most important material properties of GOES, and extensive research has been conducted since its development to reduce iron loss [3,4,5,6,7]. It is well established that the iron loss of GOES is closely related to changes in its magnetic domain structure [8,9,10]. Although the fundamental behavior of magnetic domains has been extensively investigated in nanoscale magnetic materials [11,12,13,14], the magnetic domain structure in GOES is substantially larger in scale, making theoretical analyses and computer simulations difficult even with current technologies [15,16]. Therefore, experimental investigations play a crucial role in elucidating the relationship between magnetic domain structure changes and iron loss in GOES.

In recent years, the authors have focused on a magnetic domain structure known as the spike domain [17,18,19], which forms within grains, near grain boundaries, or in regions subjected to mechanical stress, and have investigated its behavior. However, it remains unclear how the formation and annihilation of spike domains during the magnetization process affect the iron loss of GOES. As discussed above, theoretical analyses and computer simulations of spike domains are also difficult. Therefore, experimentally measuring the local iron loss is considered a practical and effective approach for elucidating the influence of spike domains on iron loss.

Conventional methods for measuring the iron loss of GOES, typified by the single sheet tester (SST) [20,21,22], evaluate the iron loss over a large area, typically 500 mm × 500 mm [23]. Consequently, these methods are incapable of assessing the influence of microscopic magnetic domain structures such as spike domains. Furthermore, the iron loss measured by the SST represents the average energy loss over one excitation cycle [23], making it impossible to evaluate the instantaneous iron loss associated with the formation and annihilation of spike domains.

Recently, the instantaneous power function has been proposed as a time-resolved representation of magnetic energy loss [24]. This function is derived from the measured waveforms of magnetic flux density, *B*, and magnetic field strength, *H* [24]. However, applications of this method have thus far been limited to measurements over large areas, such as 500 mm × 500 mm, and it remains unclear whether instantaneous iron loss can also be measured in local regions.

Several methods have been proposed for evaluating the local iron loss of GOES based on temperature rise, including infrared thermography [25,26,27] and thermistor-based measurements [28,29,30,31]. In our previous study [32], the influence of spike domains was investigated using the thermistor method. However, because of thermal diffusion, the achievable spatial resolution of the iron loss measurement was limited to approximately 10 mm × 10 mm. Furthermore, because this method relies on temperature as the measured physical quantity, it cannot evaluate instantaneous iron loss.

Methods that evaluate local iron loss using physical quantities other than temperature include needle-probe measurements [33,34,35,36,37] and Hall-effect sensor measurements [33,34,38,39,40]. Because these methods are not affected by thermal diffusion, they are promising approaches for measuring local instantaneous iron loss. However, to the best of the authors’ knowledge, no previous study has successfully measured local instantaneous iron loss using these techniques.

In this study, a method for measuring local instantaneous iron loss is proposed by combining these previous approaches. Specifically, the local waveforms of magnetic flux density, *B*, and magnetic field strength, *H*, are measured using a search coil and a Hall-effect sensor, and the local instantaneous iron loss is derived from these waveforms. As a demonstration of the proposed method, the local instantaneous iron loss in GOES before and after magnetic domain refinement is evaluated. The proposed method provides a useful tool for investigating transient local iron loss phenomena associated with magnetic domain structure changes.

## 2. Local Instantaneous Iron Loss Measurement

This section first introduces the definition of instantaneous iron loss and subsequently describes the proposed method for measuring local instantaneous iron loss.

### 2.1. Definition of Instantaneous Iron Loss

The iron loss, P (W/kg), of electrical steel is generally evaluated as the cycle-averaged value calculated from the relationship between the magnetic field strength, H(t) (A/m), and the magnetic flux density, B(t) (T), over one excitation period, T (s) [23,41,42].

(1) $$P=\frac{1}{\rho T}\int_{0}^{T}H\left(t\right)\frac{dB\left(t\right)}{dt}dt$$

where ρ (kg/$m^{3}$) is the density of the electrical steel under measurement. However, this cycle-averaged value alone does not directly provide temporal information regarding the exchange and dissipation of magnetic energy during the magnetization process. Therefore, focusing on the integrand in Equation (1), the instantaneous power function, p(t) (W/kg), is defined as follows [24]:

(2) $$p\left(t\right)=\frac{1}{\rho }H\left(t\right)\frac{dB\left(t\right)}{dt}=p_{L}\left(t\right)+p_{P}\left(t\right)$$

Here, $p_{L}\left(t\right)$ and $p_{P}\left(t\right)$ denote the loss power function and the potential energy power function, respectively, following the definition in [24]. Since the measurement position investigated in this study has a crystal orientation very close to the ideal Goss orientation, the contribution of $p_{P}\left(t\right)$ is considered sufficiently small, and $p(t)\approx p_{L}\left(t\right)$ is assumed. Therefore, p(t) is hereafter referred to as the instantaneous iron loss.

According to Equation (2), the instantaneous power function can be derived from the time-dependent waveforms of the magnetic field strength, H(t), and magnetic flux density, B(t), at the measurement location. Therefore, by obtaining the temporal variations in the local H(t) and B(t) within the target region, the local instantaneous iron loss can be evaluated. Unlike the empirical Steinmetz equation [41], which represents iron loss as a function of magnetization frequency and peak magnetic flux density, Equation (2) directly evaluates p(t) from the local H(t) and B(t), with the frequency dependence implicitly included in these waveforms.

### 2.2. Measurement of Local B(t) and H(t) Waveforms

This section describes the methods used to obtain the local magnetic flux density waveform, B(t), and the local magnetic field strength waveform, H(t).

**Figure 1:**
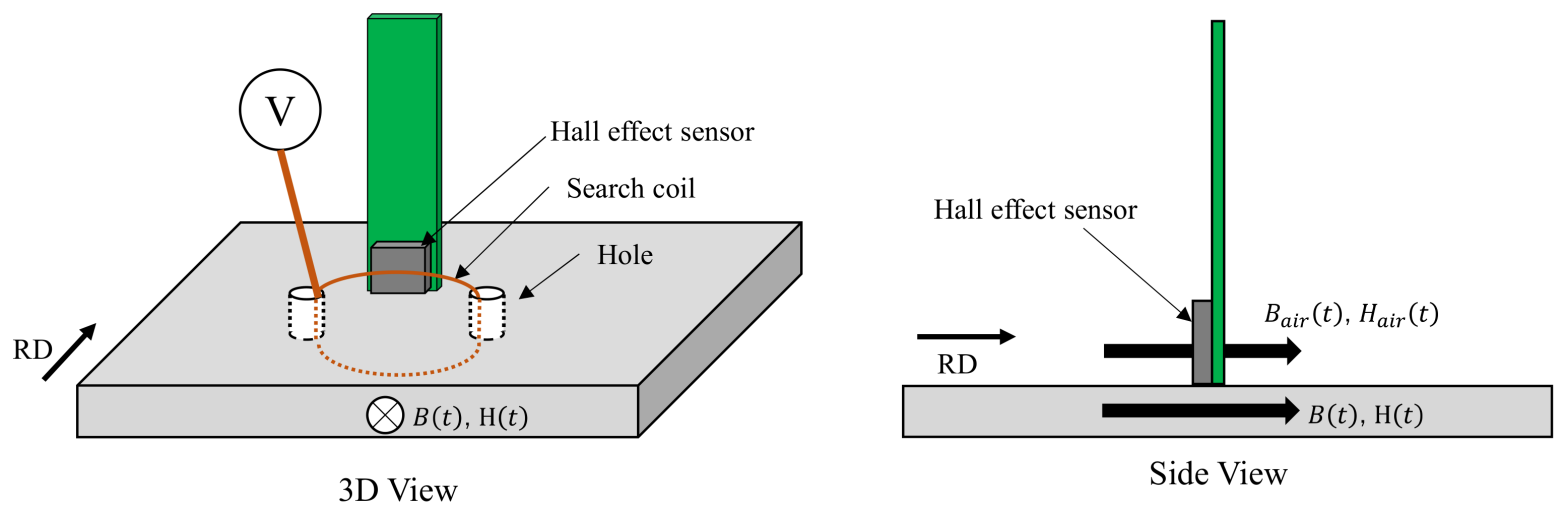
Measurement Methods for Local B(t) and H(t).

The local B(t) waveform can be measured using a needle probe [33,34,35,36,37] or a search coil. In this study, the search-coil method illustrated in Figure 1 was employed. The rolling direction (RD) of the GOES is indicated in Figure 1.

As shown in Figure 1, two holes were drilled near the region where the local magnetic flux density waveform, B(t), was to be measured, and a search coil was wound around the enclosed area. During magnetization, the local magnetic flux density waveform, B(t), was obtained from the induced voltage, $V_{s}\left(t\right)$, as follows:

(3) $$B\left(t\right)=-\frac{1}{{NA}_{t}}\int V_{s}(t)dt$$

where N is the number of turns of the search coil and $A_{t}$ ($m^{2}$) is its cross-sectional area.

When the magnetic flux density is spatially uniform over the specimen, the global magnetic flux density waveform measured using a conventional B-coil can also be used to evaluate the local instantaneous iron loss. However, when spatial variations in the magnetic flux density may exist, measuring the local B(t) corresponding to the local H(t) measurement region is preferable. Therefore, in this study, both B(t) and H(t) were measured locally to account for possible spatial variations in the magnetic flux density of GOES.

The local magnetic field strength waveform, H(t), can be measured using an H-coil [21,43,44] or a Hall-effect sensor [33,34,38,39,40]. An H-coil offers the advantages of a relatively simple measurement configuration and low sensitivity to thermal drift and static magnetic fields, such as the geomagnetic field. However, the voltage induced in an H-coil is proportional to the time derivative of the magnetic field, the cross-sectional area of the coil, and the number of turns. Consequently, when a low-frequency magnetic field is measured over a small local region, the induced voltage becomes extremely small. Increasing the coil area or the number of turns is therefore necessary to obtain a sufficiently large output voltage, making it difficult to achieve high spatial resolution simultaneously.

In contrast, a Hall-effect sensor directly converts magnetic flux density into an electrical voltage and is suitable for measurements over a small local region. Therefore, a Hall-effect sensor was employed in this study to measure the local magnetic field strength waveform, H(t).

As illustrated in Figure 1, when the magnetic flux density $B_{air}\left(t\right)$ is applied along the sensitive axis of the Hall-effect sensor, the Hall voltage, $V_{H}\left(t\right)$, is generated. The sensitivity of the Hall-effect sensor, $S_{H}$, is defined as

(4) $$S_{H}=\frac{V_{H}(t)}{B_{air}(t)}.$$

The actual output voltage of the Hall-effect sensor, $V_{out}\left(t\right)$, contains both the Hall voltage and an offset voltage, $V_{off}$, arising from sources such as the geomagnetic field and the intrinsic offset of the Hall-effect sensor. Therefore, the output voltage is expressed as

(5) $$V_{out}\left(t\right)=V_{H}\left(t\right)+V_{off}.$$

The magnetic flux density waveform, $B_{air}\left(t\right)$, can then be obtained from $V_{out}\left(t\right)$ by removing the offset voltage as

(6) $$B_{air}\left(t\right)=\frac{V_{out}\left(t\right)-V_{off}}{S_{H}}.$$

Accordingly, the magnetic field strength waveform in air, $H_{air}\left(t\right)$, is given by

(7) $$H_{air}\left(t\right)=\frac{1}{\mu_{0}S_{H}}\left(V_{out}\left(t\right)-V_{off}\right).$$

As illustrated in Figure 1, when the Hall-effect sensor is placed sufficiently close to the GOES surface, the measured magnetic field strength in air can be approximated as the magnetic field strength at the steel surface, i.e., $H_{air}\left(t\right)\approx H\left(t\right)$. This surface magnetic field should be distinguished from the applied magnetic field, since the two may differ owing to effects such as demagnetization [44].

Defining $1/\left(\mu_{0}S_{H}\right)$ as the conversion coefficient between the Hall-sensor output voltage and the alternating magnetic field, $k_{h}$, and $V_{off}/\left(\mu_{0}S_{H}\right)$ as the magnetic-field offset, $H_{off}$, the local magnetic field strength waveform of the GOES can be expressed as

(8) $$H\left(t\right)=k_{h}V_{out}\left(t\right)-H_{off}.$$

Because both the conversion coefficient, $k_{h}$, and the offset, $H_{off}$, depend on the individual Hall-effect sensor, calibration is required for each sensor. Finally, the local instantaneous power function, p(t), was obtained by substituting the local magnetic flux density waveform, B(t), obtained from Equation (3), and the local magnetic field strength waveform, H(t), obtained from Equation (8), into Equation (2).

## 3. Experimental System

This section describes the experimental system used in this study. An overview of the measurement setup is first presented, followed by detailed descriptions of each component.

**Figure 2:**
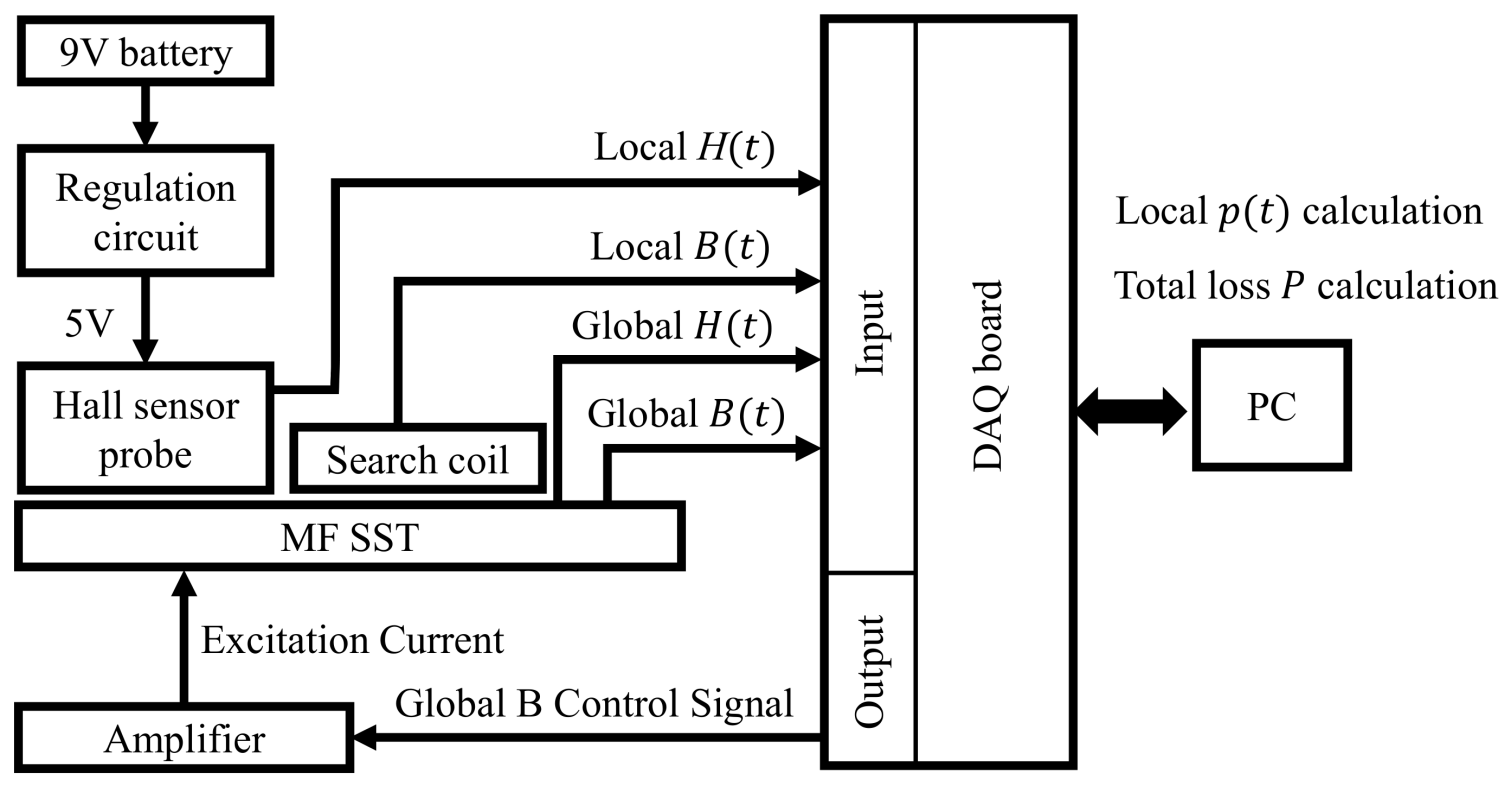
Block Diagram of the Experimental System.

Figure 2 shows a block diagram of the experimental system. A data acquisition (DAQ) board (USB-6210, manufactured by National Instruments, Austin, TX, USA) was used to control the excitation waveform and acquire the experimental data. A windowed SST (MF-SST, manufactured by Vienna Magnetics GmbH, Vienna, Austria) [45] was used to magnetize the GOES specimen. A Hall-effect sensor probe (EQ-730L, manufactured by Asahi Kasei Microdevices, Tokyo, Japan) and a search coil were positioned in the experimental window to acquire the local magnetic field waveform, H(t), and the local magnetic flux density waveform, B(t), respectively. The acquired data were transferred to a personal computer via a USB cable for subsequent signal processing.

### 3.1. SST with an Experiment Window

**Figure 3:**
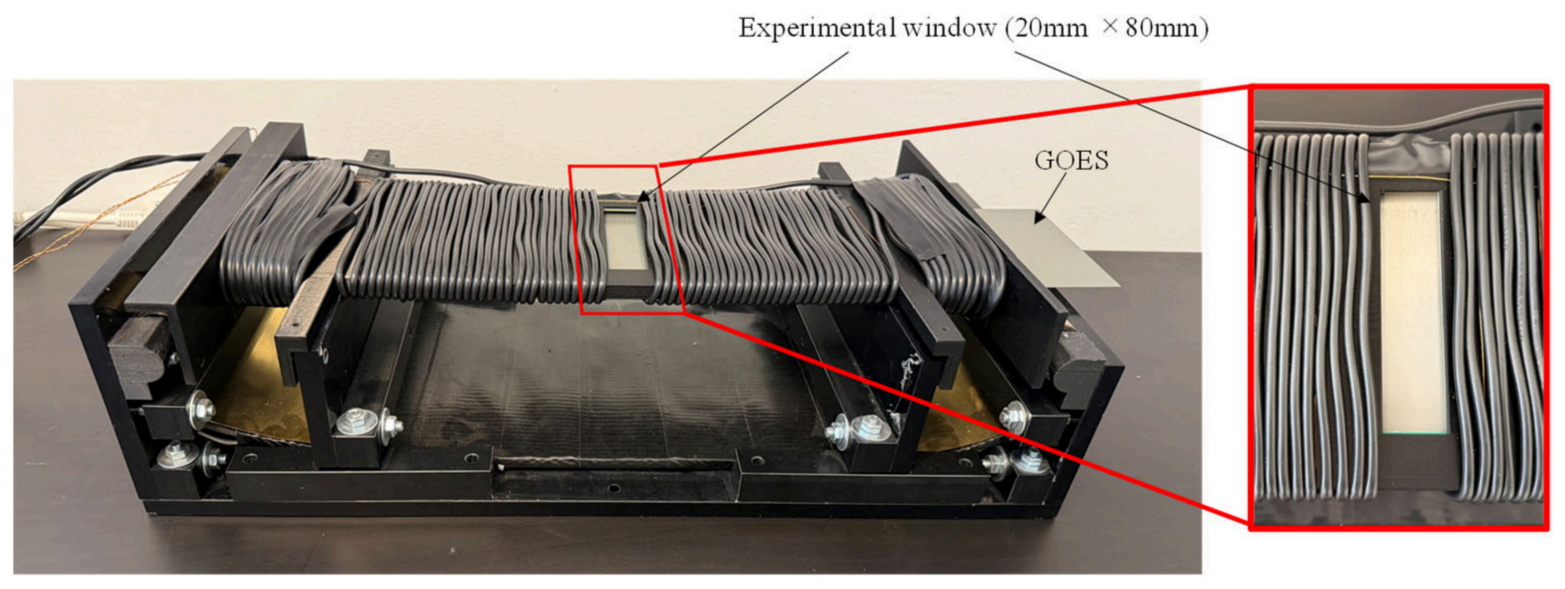
Single sheet tester (SST) with an experimental window.

The GOES specimen was magnetized using the SST with an experimental window, as shown in Figure 3 [45].

Compared with a conventional SST, this system employs a reduced specimen size (500 mm × 100 mm), an amorphous single yoke suitable for magnetization up to 10 kHz, two main coils and two booster coils for improved spatial induction control, and printed tangential H-coils for direct magnetic field measurement. The repeatability of the global iron loss measurement is within 0.3%, and the absolute accuracy is within 2% [46].

The principal feature of this system is that an experimental window is provided at the center of both the B-coil and the H-coil [45]. By placing the Hall-effect sensor probe and the search coil inside the experimental window, the local iron loss generated during the magnetization of the GOES specimen can be measured under a relatively uniform magnetic field while simultaneously measuring the global iron loss of the entire sheet.

### 3.2. Hall-Effect Sensor Probe

**Figure 4:**
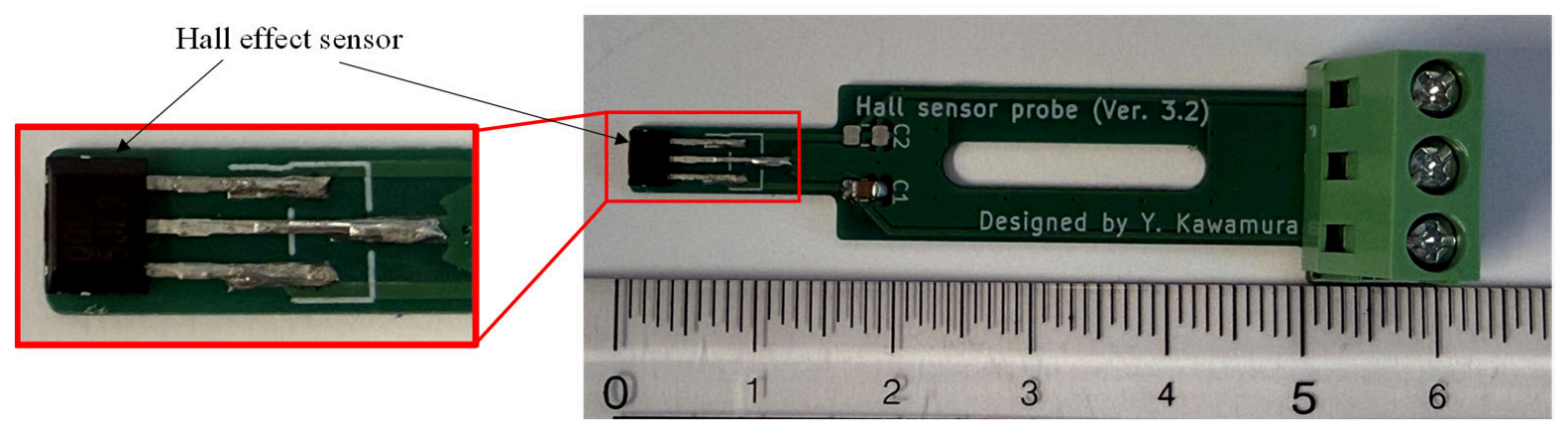
Photograph of the Hall-effect sensor probe.

The Hall-effect sensor probe shown in Figure 4 was used to measure the local magnetic field waveform, H(t).

The local magnetic field waveform, H(t), was obtained by multiplying the output voltage of the Hall-effect sensor probe, $V_{out}\left(t\right)$, by the conversion coefficient $k_{h}$ and subtracting the offset. Since the conversion coefficient $k_{h}$ depends on the characteristics of the Hall-effect sensor probe, it was determined by calibration using a solenoid coil.

For the calibration, the Hall-effect sensor probe was positioned at the center of a cylindrical solenoid coil with a diameter of 0.10 m, a length of 0.67 m, and 420 turns, such that the sensitive axis of the Hall-effect sensor was parallel to the magnetic field. The output voltage, $V_{out}\left(t\right)$, was then measured under a known magnetic field.

If the conversion coefficient $k_{h}$ exhibits frequency or amplitude dependence, additional compensation is required instead of using a single constant coefficient. Therefore, the linearity of the Hall-effect sensor probe was evaluated over a frequency range from 1 Hz to 10 kHz and a magnetic field amplitude range from 3 A/m to 80 A/m. The experimental results confirmed that the Hall-effect sensor probe exhibited sufficient linearity within these ranges, and the conversion coefficient was identified as $k_{h}=5935$ A/(m·V). Based on the operating range of the MF-SST and the experimentally evaluated frequency range of the Hall-effect sensor probe, the practical upper frequency of the present setup is considered to be approximately 10 kHz.

### 3.3. Signal Processing for Noise and Offset Compensation

The magnetic flux density waveform, B(t), and the magnetic field waveform, H(t), obtained from Equations (3) and (8), respectively, contain measurement noise and offset components. Therefore, signal processing was performed to improve the signal quality before calculating the instantaneous iron loss. The signal processing procedure used in this study is described below.

The GOES specimen was magnetized by the SST using a sinusoidal excitation with a period of T. Accordingly, both the magnetic flux density waveform and the magnetic field waveform are periodic with the same period. Let the raw magnetic flux density waveform and the raw magnetic field waveform immediately after measurement be denoted as $B_{raw}\left(t\right)$ and $H_{raw}\left(t\right)$, respectively.

To reduce measurement noise, the raw waveforms were synchronized with the excitation period T and averaged over M cycles according to

(9) $$x_{ave}\left(t\right)=\frac{1}{M}\sum_{k=0}^{M-1}x_{raw}\left(t+kT\right)$$

where 0≤t<T.

Next, because the averaged magnetic field waveform, $H_{ave}\left(t\right)$, may still contain a DC offset, the offset $H_{off}$ was estimated from the mean values within the intervals of ±Δt around t=0 and t=T/2, as

(10) $$H_{off}=\frac{1}{4∆t}\int_{-∆t}^{+∆t}H_{ave}\left(t\right)+H_{ave}\left(t+\frac{T}{2}\right)dt,$$

The offset estimation in Equation (10) allows the magnetic field waveform to be processed either with offset compensation alone or with the symmetrization procedure described below. When symmetrization is not applied, the offset-compensated magnetic field waveform is given by

(11) $$H_{ave}^{off}\left(t\right)=H_{ave}\left(t\right)-H_{off}.$$

In this study, the magnetic field waveform was further symmetrized to suppress residual DC magnetic-field components, such as those caused by the Earth’s magnetic field, and their influence on the slight DC magnetization of the GOES specimen. Assuming half-cycle antisymmetry under sinusoidal magnetization, the symmetrized waveform was calculated as

(12) $$H_{ave}^{sym}\left(t\right)=\frac{H_{ave}\left(t\right)-H_{ave}\left(t+\frac{T}{2}\right)}{2},\;\;\;0\leq t<\frac{T}{2}.$$

The symmetrized waveform for the second half-cycle was defined by

(13) $$H_{ave}^{sym}\left(t+\frac{T}{2}\right)=-H_{ave}^{sym}\left(t\right).$$

Because the DC offset is canceled by the half-cycle subtraction in Equation (12), $H_{off}$ does not appear explicitly in the symmetrized waveform.

In this study, the averaged waveform $B_{ave}\left(t\right)$, obtained by applying Equation (9) to $B_{raw}\left(t\right)$, was defined as the local magnetic flux density waveform B(t). Similarly, the waveform $H_{ave}^{sym}\left(t\right)$, obtained by applying the cycle averaging and symmetrization procedures to $H_{raw}\left(t\right)$, was defined as the local magnetic field waveform H(t). These processed waveforms were used to calculate the local instantaneous power function p(t).

## 4. Experimental Setup

This section describes the experimental conditions. To validate the effectiveness of the proposed method for measuring local instantaneous iron loss, the instantaneous iron loss waveforms of the GOES specimen under two different conditions were measured and compared.

A GOES specimen with a thickness of 0.23 mm was used in the experiments. Before the measurements, the specimen was annealed to relieve the mechanical stress introduced by shearing and other mechanical processing.

**Figure 5:**
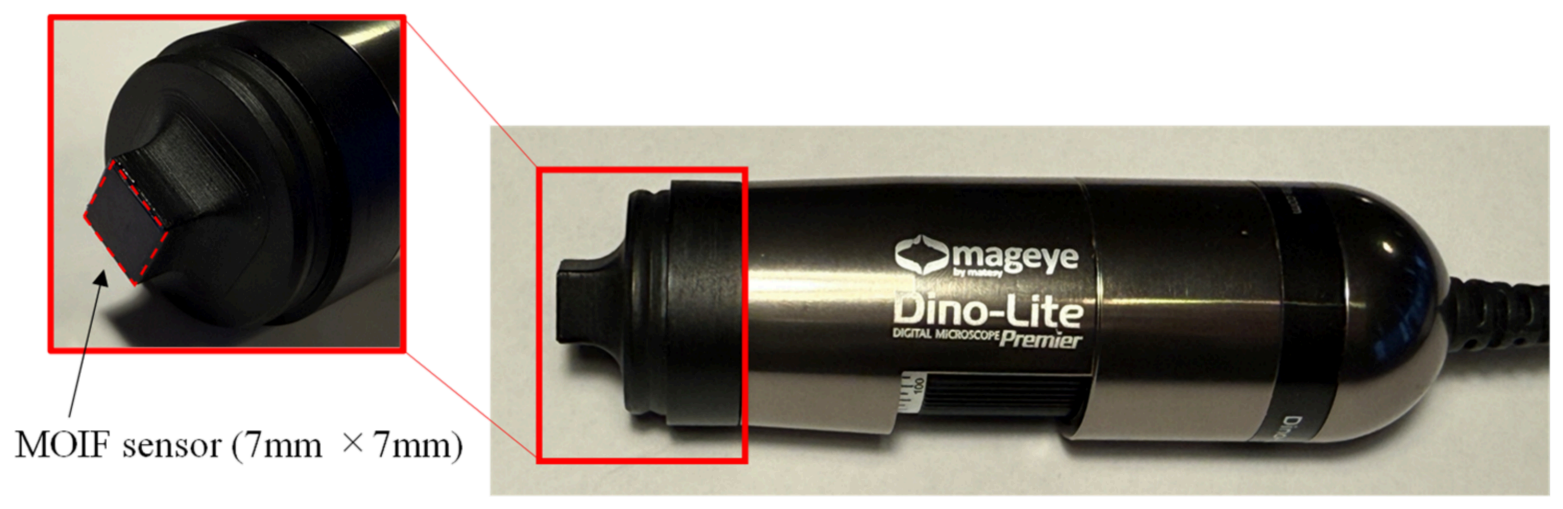
Photograph of the Mageye system.

First, the magnetic domain structure of the specimen was observed using the Faraday effect. The Mageye system shown in Figure 5 (manufactured by Matesy GmbH, Jena, Germany) was used for the observation. This system visualizes magnetic domain structures based on the Faraday effect and provides magnetic domain images over an area of approximately 7 mm×7 mm with a resolution of 2592×1944 pixels. For observations over a wider region, the contrast of the acquired images was adjusted, and multiple images were combined to form a continuous magnetic domain image.

Using the Mageye system, a region near the center of the GOES specimen with a relatively uniform magnetic domain structure was selected. Two holes with a diameter of 1 mm were drilled at a spacing of 15 mm to form a search coil. The line connecting the two holes was located approximately 5 mm outside the upper edge of the magnetic domain observation region. The holes were drilled after the stress-relief annealing, and no additional annealing was performed after drilling. The search coil consisted of one turn (N=1), and the cross-sectional area enclosed by the search coil was $A_{t}=15\;mm\times 0.23\;mm=3.45\times 10^{-6}m^{2}$. The holes may locally disturb the magnetic flux distribution in their vicinity. When such an influence is of concern and the magnetic flux density is sufficiently uniform over the specimen, the global magnetic flux density waveform measured by the SST can be used instead. The Hall-effect sensor probe was positioned at the center of the selected region, approximately 12.5 mm from the line connecting the two search-coil holes.

The GOES specimen was magnetized using the MF-SST shown in Figure 3 at a frequency of 50 Hz and a magnetic flux density amplitude of 1.7 T. The local instantaneous iron loss at the selected position was then measured.

**Figure 6:**
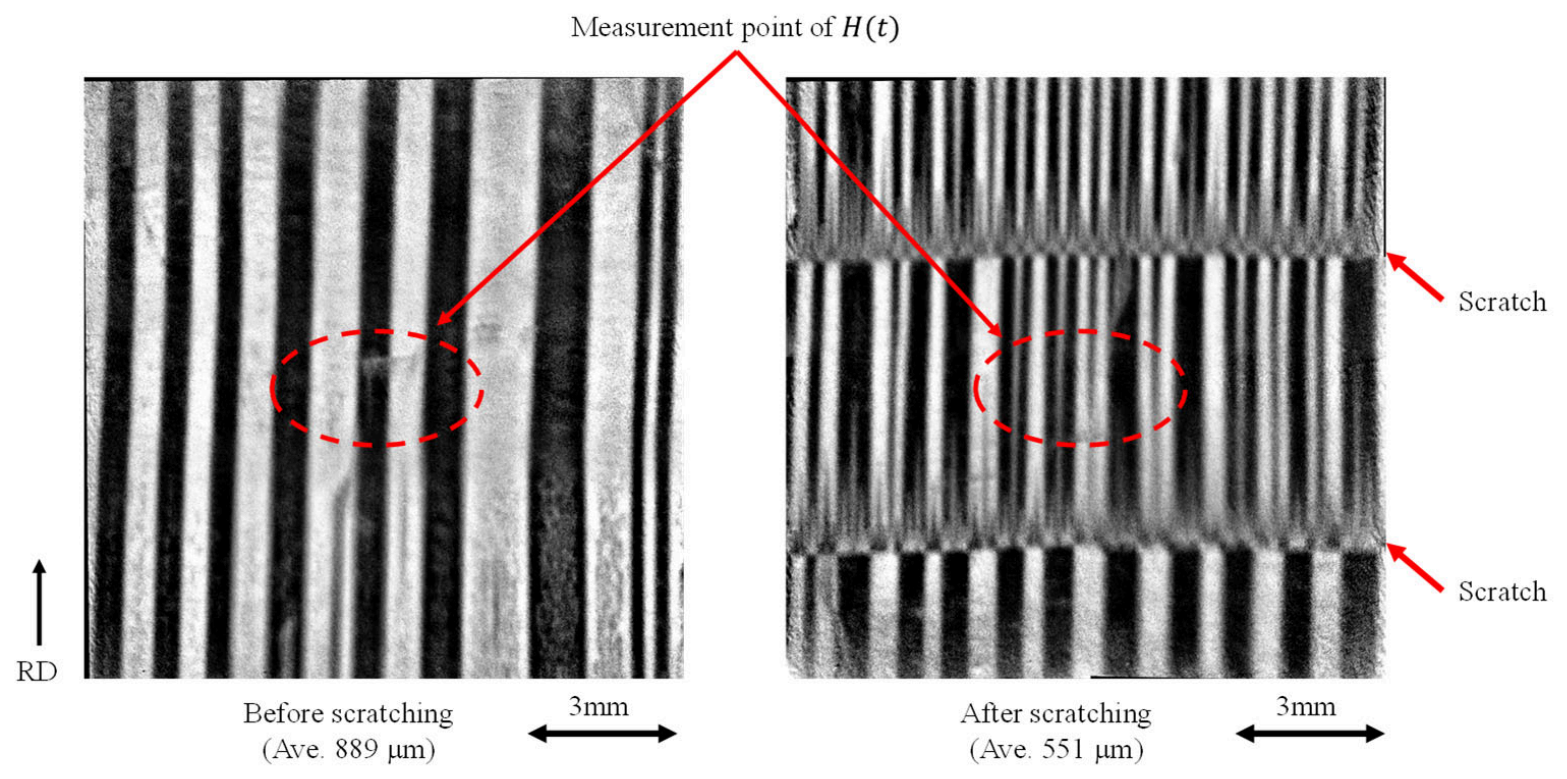
Magnetic Domain Structures Before and After Scratching.

After the first measurement, two scratches were manually introduced at a spacing of 5 mm in the transverse direction (TD), orthogonal to the rolling direction (RD), using a ballpoint pen to refine the magnetic domain structure. Scratching-induced domain refinement has been discussed in terms of mechanical stress [47,48,49], while the role of the strain field has also been investigated in [50]. Only these two scratches, as shown in Figure 6, were introduced into the specimen. Since the scratches were manually introduced using a ballpoint pen, the applied mechanical force was not precisely controlled or quantitatively measured.

The local instantaneous iron loss was then measured again at the same position. By comparing the measured local instantaneous iron loss waveforms before and after scratching, the influence of magnetic domain refinement on the local instantaneous iron loss was evaluated.

## 5. Experimental Results and Discussion

This section presents the experimental results and discusses the effectiveness of the proposed method.

### 5.1. Changes in the Magnetic Domain Structure Before and After Scratching

Figure 6 shows the magnetic domain structures at the location where the local instantaneous iron loss was measured before and after scratching.

Figure 6 shows the magnetic domain structures before and after scratching. Before scratching, a relatively uniform magnetic domain structure was observed. In contrast, after scratching, the magnetic domains were clearly refined by the two scratches introduced with the ballpoint pen.

The average 180° domain-wall spacing was quantified using the method previously proposed by the authors [51]. The average spacing decreased from 889 μm before scratching to 551 μm after scratching, corresponding to a reduction of approximately 340 μm.

### 5.2. Measurement Results for Local Instantaneous Iron Losses

**Figure 7:**
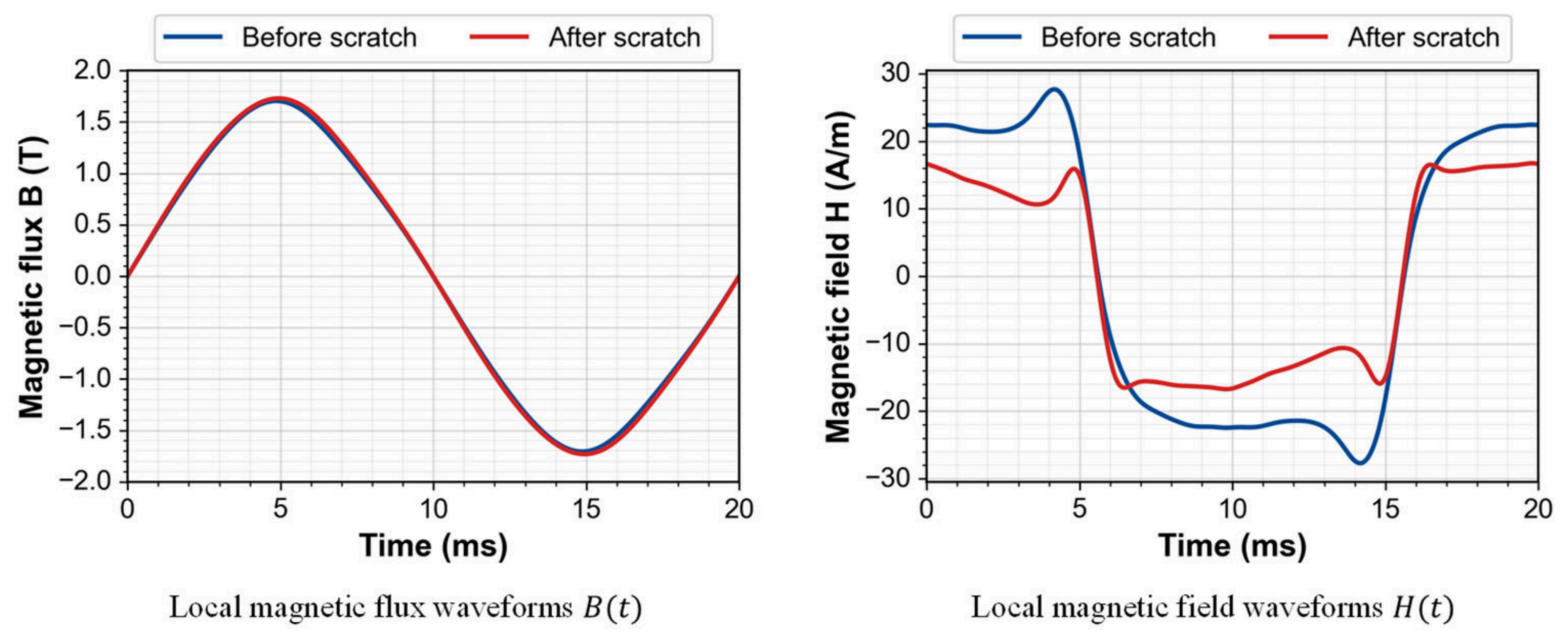
Measured waveforms of B(t) and H(t).

Figure 7 shows the local magnetic flux density waveform, B(t), measured using the search coil and the local magnetic field waveform, H(t), after the symmetrization process described in Section 3.3.

The measured local magnetic flux density waveform, B(t), shown in Figure 7 was approximately sinusoidal with an amplitude of 1.7 T at 50 Hz, indicating that the local magnetic flux density at the selected region was close to the global magnetization condition. No significant change in the local magnetic flux density waveform, B(t), was observed before and after scratching.

In contrast, the local magnetic field waveform, H(t), changed markedly after scratching. The maximum amplitude of H(t) decreased from approximately 30 A/m before scratching to approximately 20 A/m after scratching.

**Figure 8:**
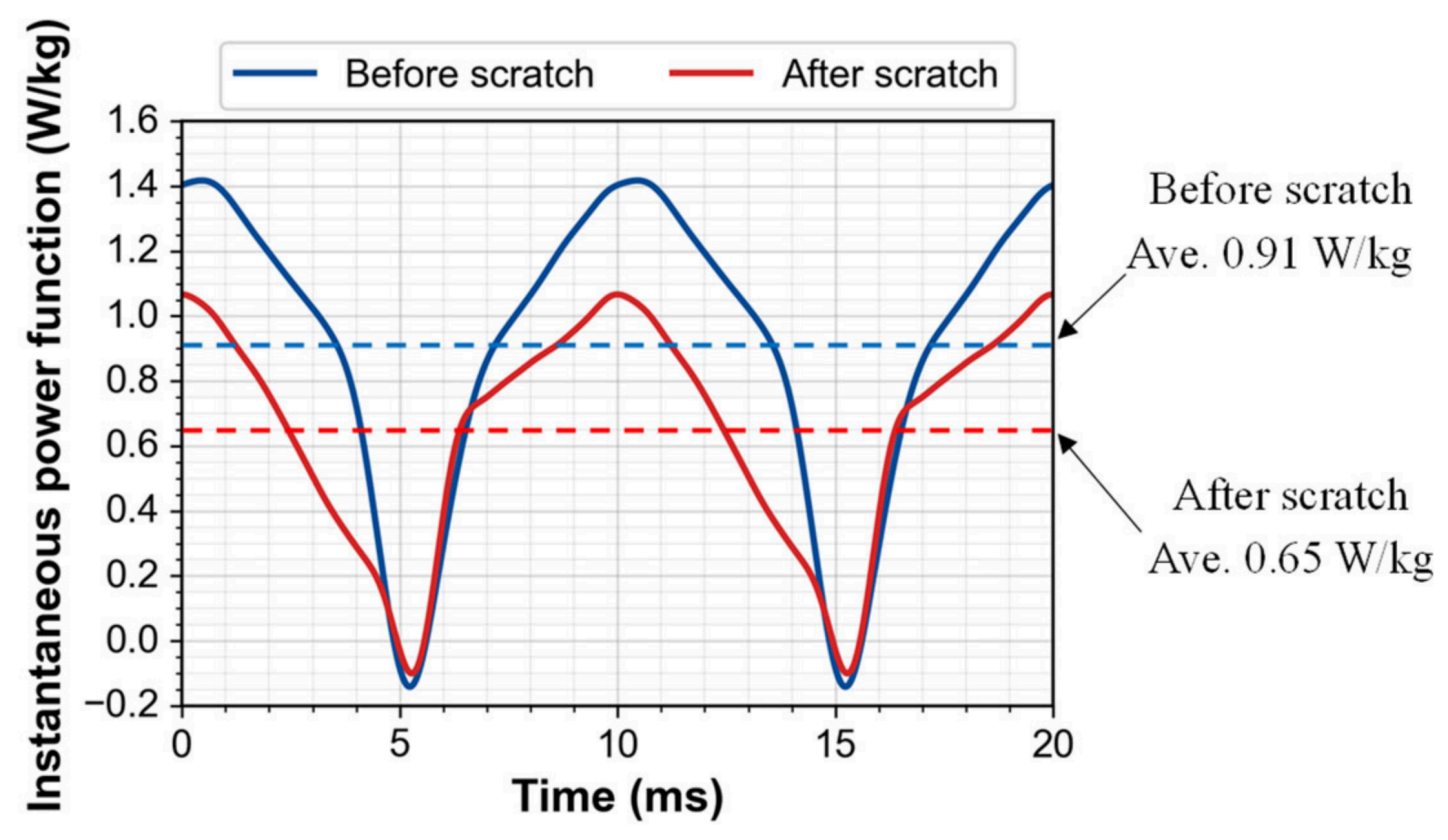
Instantaneous power function p(t) before and after scratching with a ballpoint pen.

The local instantaneous power function, p(t), was then calculated from the measured B(t) and H(t) using Equation (2). Figure 8 shows the instantaneous power function before and after scratching. As described in Section 2.1, the instantaneous power function is regarded as the local instantaneous iron loss in the present study based on the approximation $p(t)\approx p_{L}\left(t\right)$.

As shown in Figure 8, the local instantaneous iron loss was reduced over almost the entire magnetization cycle after scratching. The average iron loss over one magnetization cycle decreased from 0.91 W/kg before scratching to 0.65 W/kg after scratching, corresponding to a reduction of approximately 29%.

### 5.3. Discussion

First, the reduction in the local iron loss before and after scratching is discussed. Previous studies have reported that scratching, laser scribing, and similar domain refinement techniques reduce the average iron loss of an entire GOES sheet by approximately 10–20% [52,53,54,55]. In contrast, the reduction observed in the present study (approximately 29%) is relatively large compared with these previously reported values.

One possible explanation is that the local iron loss was measured in a region where the influence of residual mechanical stress was relatively small. The change in iron loss caused by scratching can be interpreted as the superposition of two competing effects: the reduction in iron loss due to magnetic domain refinement and the increase in iron loss caused by the residual mechanical stress introduced by scratching. The conventional average iron loss of the entire sheet includes the combined influence of both effects.

In the present study, however, the measurement position was located midway between the two scratches, where the influence of residual mechanical stress was expected to be smaller than that in the vicinity of the scratches. Consequently, the iron loss reduction due to magnetic domain refinement may have become more dominant, resulting in a larger reduction in the local iron loss than that reported for the average iron loss of the entire sheet.

The validity of the measured local iron loss was further assessed by comparison with the previous study [47]. The measurement location selected in the present study exhibited relatively wide magnetic domains, and the 180° domain walls were almost parallel to the rolling direction. Based on the observed magnetic domain structure, the crystal orientation at this location is considered to be close to the ideal Goss orientation, with an *α* angle of approximately 0° and a *β* angle of approximately 0–1°.

The previous study [47] reported the iron loss of a 0.20 mm thick single crystal for various crystal orientations and 180° domain-wall spacings. Under conditions comparable to those after scratching in the present study, the reported iron loss was approximately 0.5–0.6 W/kg. Although the specimen thickness used in the present study was slightly larger (0.23 mm), the measured local iron loss of 0.65 W/kg is considered to be reasonably consistent with the previous results, taking into account the increase in eddy-current loss associated with the greater sheet thickness. Therefore, the measured local iron loss obtained using the proposed method is considered to be reasonable.

Next, attention is focused on the local instantaneous iron loss before and after scratching. The slightly negative values of p(t) originate from the negative contribution of the potential energy power function, $p_{P}\left(t\right)$, associated with reversible magnetization processes and do not represent negative energy dissipation [24]. A noticeable difference in the local instantaneous iron loss was observed between approximately 7 ms and 14 ms. According to the local magnetic flux density waveform shown in Figure 7, this time interval corresponds to a local magnetic flux density ranging from approximately +1.2 T to −1.6 T.

Under sinusoidal magnetization with a magnetic flux density amplitude of 1.7 T, this flux density range is known to correspond to the region where magnetic domain-wall motion is most active and, consequently, where anomalous eddy-current loss associated with domain-wall motion becomes significant. Therefore, the reduction in the local instantaneous iron loss in this region after scratching is consistent with the refinement of the magnetic domains.

In contrast, little difference in the local instantaneous iron loss waveform was observed outside the flux density range from +1.2 T to −1.6 T. This result suggests that magnetic domain refinement has only a limited influence on the local iron loss in these regions.

When the same experiment is performed using a conventional SST or a conventional local iron loss measurement method, it can only be concluded that the average iron loss decreases after scratching. In contrast, the proposed method provides not only the conventional evaluation of the iron loss reduction but also reveals that magnetic domain refinement contributes to iron loss reduction only within a specific magnetic flux density range, while having little influence in other regions. To the best of the authors’ knowledge, this information cannot be obtained using conventional iron loss evaluation methods.

At present, the proposed method has been demonstrated only for the evaluation of the effect of magnetic domain refinement. However, as described in the Introduction, the proposed method is also applicable to the evaluation of iron loss changes associated with the generation and disappearance of fine magnetic domain structures, such as spike domains.

## 6. Conclusions

In this paper, a measurement method using a Hall-effect sensor was proposed for measuring the local instantaneous iron loss in GOES. To verify the effectiveness of the proposed method, the local instantaneous iron loss was measured at the same location before and after scratching. The experimental results showed that the average 180° domain-wall spacing decreased by approximately 340 μm after scratching. The measured local iron loss after scratching was also found to be reasonably consistent with previously reported values for single crystals under comparable conditions, supporting the validity of the proposed measurement method. Furthermore, magnetic domain refinement reduced the local instantaneous iron loss. The reduction in the local instantaneous iron loss was observed mainly within the magnetic flux density range where domain-wall motion is most active, which is physically consistent with the mechanism of anomalous eddy-current loss. In contrast, other magnetic flux density regions exhibited little change in the local instantaneous iron loss after domain refinement. These results reveal that the contribution of magnetic domain refinement to iron loss reduction depends on the magnetic flux density region.

Compared with conventional SST measurements, the proposed method significantly improves both the temporal and spatial resolution of iron loss evaluation in GOES. In the future, by combining the proposed method with dynamic magnetic domain observation, it will be possible to investigate the local generation of iron loss associated with changes in magnetic domain structures in much greater detail than has previously been possible. Such analyses are expected to provide new insights into the mechanisms of iron loss and may contribute to further optimization of magnetic domain refinement techniques for reducing the iron loss of GOES.

## Data Availability

The data presented in this study are available from the corresponding author upon reasonable request. The data are not publicly available due to proprietary and confidentiality restrictions associated with the materials and experimental setup.

## Abbreviations

The following abbreviations are used in this manuscript:

GOES	-	grain-oriented electrical steel
SST	-	single sheet tester
RD	-	rolling direction
TD	-	transverse direction
P	W/kg	one-cycle averaged iron loss
ρ	kg/m^3^	density of the specimen
t	s	time
f	Hz	magnetization frequency
T	s	magnetization period
H(t)	A/m	local magnetic field waveform
B(t)	T	local magnetic flux density waveform
p(t)	W/kg	local instantaneous power function (≈local instantaneous iron loss)
$p_{L}(t)$	W/kg	local instantaneous loss power function
$p_{P}(t)$	W/kg	local instantaneous potential energy power function
*N*	-	number of turns of the search coil
$A_{t}$	m^2^	cross-sectional area enclosed by the search coil
$V_{s}(t)$	V	search-coil voltage
$S_{H}$	V/T	Hall-sensor sensitivity
$V_{H}(t)$	V	Hall voltage
$B_{air}(t)$	T	magnetic flux density in the air above the GOES surface
$V_{out}(t)$	V	Hall-sensor output voltage
$V_{off}$	V	Hall-sensor offset voltage
$H_{air}(t)$	A/m	magnetic field in the air above the GOES surface
$k_{h}$	A/(m·V)	conversion coefficient from Hall voltage to magnetic field
$H_{off}$	A/m	magnetic-field offset
$B_{raw}\left(t\right)$, $H_{raw}\left(t\right)$	T, A/m	measured raw B and H waveforms
$B_{ave}(t)$, $H_{ave}\left(t\right)$	T, A/m	synchronized and averaged B and H waveforms
$H_{ave}^{off}\left(t\right)$	A/m	synchronized and averaged H waveform after offset removal
$H_{ave}^{sym}\left(t\right)$	A/m	synchronized, averaged, and symmetrized H waveform
